# High‐throughput sequencing‐based analysis of fungal diversity and taste quality evaluation of Douchi, a traditional fermented food

**DOI:** 10.1002/fsn3.1953

**Published:** 2020-10-27

**Authors:** Yurong Wang, Fanshu Xiang, Zhendong Zhang, Qiangchuan Hou, Zhuang Guo

**Affiliations:** ^1^ Hubei Provincial Engineering and Technology Research Center for Food Ingredients Hubei University of Arts and Science Xiangyang China

**Keywords:** Douchi, electronic tongue, fermented soybean, fungal diversity

## Abstract

Douchi, a popular traditional fermented soybean product, is mainly made by natural fermentation. However, its taste quality is affected by specific fungal communities which vary greatly according to fermentation conditions and production technologies used in different regions. Therefore, the taste quality of Douchi samples from different regions was digitally evaluated using electronic tongue technology. In addition, the fungal community structures and its association of them were also identified using high‐throughput sequencing technology. Results showed that there were obvious differences in the taste quality of samples from different regions, while the tastes of different types of samples from the same region were similar. Sourness, umami, richness, and saltiness were the main reasons for regional differences in taste. Similarly, the results of high‐throughput sequencing indicated that samples from different regions displayed important differences in dominant fungal genus, among which *Debaryomyces*, *Fusarium*, *Pichia*, *Aspergillus,* and *Saccharomyces* were the main dominant fungi. *Debaryomyces* and *Trichosporon* were conducive to the formation of taste qualities of Douchi, while *Cladosporium* and *Candida* have a negative impact on the taste quality of Douchi var correlation analysis. This study indicated the effects of dominant fungi on the formation of Douchi taste quality, allowing a deeper understanding of the role of microbial species in generating fermented soybean products in China. Our work provides theoretical support to guide the improvement of the industrial production process of Douchi.

## Graphical abstract

- *Debaryomyces*, *Fusarium*, *Pichia*, *Aspergillus*, and *Saccharomyces* were the main dominant fungi of Douchi.
- *Debaryomyces*, *Fusarium*, *Pichia*, *Aspergillus*, and *Saccharomyces* led to important differences in the overall fungal structure of samples from different groups.
- Sourness, umami, richness, and saltiness were the main reasons for regional differences in taste.
- *Debaryomyces* and *Trichosporon* were conducive to the formation of taste qualities of Douchi, while *Cladosporium* and *Candida* have a negative impact on the taste quality of Douchi.

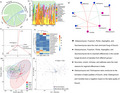

## 1 INTRODUCTION

Fermented bean products have a long history in China. Douchi (also known as "You Shu" in ancient times) is not only one of the most popular fermented bean products in China but was also the main seasoning in ancient times (Chen et al., 2007). Douchi is made using soybeans or black beans as the main raw materials, supplemented with salt, liquor, and various spices. The special taste and flavor of Douchi are produced during the decomposition of soybean proteins through fermentation by *Aspergillus*, *Mucor*, or by *proteases* (Wu, 2011; Hu H. 2014 ). While there are many kinds of Douchi in China, they can be separated into dried Douchi and wet Douchi (natto), the main difference relating to the amount of water added during fermentation. In recent years, related studies have proved that traditional fermented soybean products have several health benefits, including reducing the number of inflammatory factors in the body (Jung et al., 2016 ), promoting appetite (Reseland et al., 2001), preventing the formation of cervical thrombus (Yuan et al., 2012), and improving the structure of intestinal flora (Dong et al., 2014). Therefore, Douchi is favored by consumers due to its unique taste as well as its nutritional characteristics.

The formation of taste, flavor, and texture of fermented bean products is closely related to the growth and reproduction of fungi during the process of fermentation, because they produce various amino acids by decomposing proteins, which plays an important role in the generation of flavor compound in Douchi (Hu et al., 2013). In recent years, an increasing number of studies have analyzed the composition and structure of the microbial community in fermented soybean products (Chen et al., 2011; Tamang et al., 2016). Previous studies mainly relied on culture‐dependent method to identify microorganisms in fermented soybean products. For instance, it was found that the number of microorganisms on the surface of Douchi was 10‐ to 100‐fold lower compared with the middle and lower parts of Douchi. On the surface of Douchi, the main species were found to be *Lactococcus lactis* and *Bacillus thermoamylovorans*, while *Staphylococcus lentus* and 2 uncultured strains occupied the dominant positions below the surface. The denaturing gradient gel electrophoresis patterns of the fungi showed that the structure of the fungal community on the surface and inside Douchi was similar and was mainly composed of yeast (Chen et al., 2012). In addition, the results of high‐throughput sequencing showed that the main fungi in Douchi were *Pichia* and *Candida*. On the other hand, five kinds of bacteria related to Douchi fermentation have been identified including *Rhodosporidium*, *Yarrowia*, *Aureobasidium*, *Aspergillus*, and *Dipodascus* (Chen et al., 2014). While the above studies provide some reference information for exploring the microbial diversity in Douchi, there is still a lack of a research to compare the overall differences of fungal communities among different types of Douchi. In addition, the unique taste and community structure of fermented soybean products are also affected by the region (Zhang et al., 2018). Although some studies have been carried out on the microbial diversity of Douchi in China, few have dealt with the relationship between microbial diversity and taste quality. A key reason was the lack of necessary technical means to determine the relationship between the type of microorganisms and the quality of fermented soybean products effectively and digitally.

In recent years, the second‐generation sequencing technology represented by Illumina MiSeq has been developing rapidly. Compared with traditional molecular biology methods, high‐throughput sequencing technology can not only quickly and accurately analyze the microorganisms in traditional fermented food, but also provide more detailed information and more in‐depth research on microbial diversity (Schirmer et al., 2015; Roh et al., 2010). Meanwhile, the rapid rise of electronic tongue technology, which not only eliminates the influence of subjective factors but also quickly and digitally evaluates the taste index, has shown promise in the field of traditional fermented foods including the digital evaluation of Douchi.

In the present study, MiSeq high‐throughput sequencing technology was used to identify the fungal communities in two types of Douchi from two regions and was combined with electronic tongue technology to evaluate their taste quality digitally. The differences of fungal community structure and product quality in different types of fermented soybeans from two regions were systematically analyzed, and the influence of the former on the latter was further explored. The purpose of this work was to deepen our understanding of fungal microorganisms in Douchi, and to explore their effects on the taste and quality of Douchi, with the goal of improving the fermentation process and industrial production of Douchi.

## 2 MATERIALS AND METHODS

### 2.1 Sample collection

Thirty‐nine Douchi samples were collected from Enshi Tujia and Miao Autonomous Prefecture, Hubei Province, China. Among them, nineteen were collected in Lichuan County (E108°21′–109°18′, N29°42′–30°39′) (10 of dried Douchi, samples LCD1–LCD10, and 9 of wet Douchi, samples LCW1–LCW9), and 20 Douchi samples were collected in Badong County (E110°04′–110°32′, 30°28′–31°28′) (11 dried Douchi: BDD1–BDD11, and 9 wet Douchi: BDW1–BDW9). All samples were collected from local farmers' home. The main raw materials were soybeans, and the auxiliary materials were salt, pepper, ginger, and so on. Cook soybeans, spread them out and cool them, add auxiliary materials, air them outdoors until they are semi‐dry, and then put them in clay pots for natural fermentation. Wet fermented soybeans will be fermented together with some boiled soybean soup when entering the altar. The average moisture content of dry Douchi in this study was 11.29%, while that of wet Douchi was 57.56%. Samples were picked up by a sterile spoon, placed into a special aseptic sample bag, and transported back to the laboratory. Samples were stored at −4°C until use.

### 2.2 Determination of moisture content and taste quality

The moisture content was determined according to the direct drying method in GB/T 5009.3‐2010 "Determination of moisture in food" (Thuwapanichayanan et al., 2011). Briefly, a clean, flat glass weighing bottle was placed in a drying box at 100°C for 1 hr and cooled in a desiccator for 0.5 hr before weighing, and this was repeated until constant weight was obtained. Next, ground Douchi (10 g) was placed in the weighing bottle, covered, closed tightly, and placed in a drying box at 100°C for 2–4 hr. After that, the dried Douchi was cooled in a desiccator for 0.5 hr and then weighed. Then, the sample was placed again in a drying box at 100°C for about 1 hr, cooled in a desiccator for 0.5 hr, and weighed again. When the difference between two subsequent measurements was less than 2 mg, the product was considered at constant weight.

After grinding the collected samples, 20 g was added to 100 ml distilled water and stirred and soaked for 2 hr. Then, the mixture was centrifuged at 10,000 r/min for 10 min, and the supernatant was refrigerated in a measuring cylinder at 4°C for 24 hr, and the oil layer was removed (Dong et al., 2019). After the sensor self‐test and diagnosis in the electronic tongue system, the Douchi samples that had been treated with 100 ml were evenly poured into two sample test cups, and the basic tastes such as sourness (CAO), bitterness (COO), astringency (AE1), saltiness (CTO), and umami (AAE) were determined, as well as aftertaste‐A, aftertaste‐B, and richness. Each Douchi sample was analyzed 4 times, and the measured data of the last 3 times were selected for follow‐up analysis (Zhao et al., 2016).

### 2.3 DNA extraction, PCR amplification, and pyrosequencing

After the Douchi sample was ground evenly, 5 g was added to 45 ml normal saline. After beating for 10 min with a beater and centrifugation at 400 r/min for 5 min, the supernatant was again centrifuged at 10,000 r/min for 10 min. The macrogenomic DNA was extracted from the precipitate by QIAGEN DNeasy Mericon Food Kit, and the DNA samples were stored at −20°C (Wang et al., 2018).

The extracted macrogenomic DNA was amplified by PCR using the forward primer SSU0817F (5'‐TTAGCATGGAATAATRRAATAGGA‐3') and the reverse primer SSU1196R (5'‐TTAGCATGGAATARAATAGGAMAULY‐3'). Furthermore, seven nucleotide tags were added to the forward primer (Lin, 2019). The PCR amplification system was as follows: 0.8 μL forward primer (5 μmol/L), 0.8 μl reverse primer (5 μmol/L), 2 μl dNTPs mix (2.5 mmol/L), 4 μl 5× PCR buffer, 10 ng DNA template, and 0.4 μl DNA polymerase (5 U/μl). The mix was supplemented with ddH_2_O to 20 μl. The PCR amplification conditions were as follows: 95°C 3 min, 95°C 30 s, 55°C 30 s, 72°C 45 s, 30 cycles; final extension at 72°C for 10 min (Tall & Meyling, 2018). The DNA products were sequenced by Illumina MiSeq high‐throughput sequencing platform.

### 2.4 Bioinformatic analysis

According to the overlap of the two‐terminal sequences, the offline data were spliced into a single sequence and the spliced sequence was distributed according to the barcode information. Finally, the primer and barcode information were removed. After quality control, the QIIME (v 1.70) platform was used to analyze the species composition and fungal diversity. The main analysis process was as follows (Yang et al., 2020): (a) PyNAST software was used for standard alignment and alignment of effective sequences after quality control (Caporaso et al., 2010). (b) After dividing the aligned sequences according to 100% similarity, a classification Operational Taxonomic Unit (OTU) was constructed according to 97% similarity (Edgar, 2013). (c) ChimeraSlayer software was used to identify and delete the chimera sequences in the OTU sequences (Edgar et al., 2011). (d) Homology alignment was performed on the first sequence (representative sequence) in each OTU, and the taxonomic status of each OTU at the level of phylum, class, order, family, and genus was obtained (Quast et al., 2012; Yilmaz et al., 2014). (e) A phylogenetic tree was built through FastTree software, and on this basis, the alpha diversity index of the microbial flora in the Douchi samples was calculated, to evaluate the diversity and abundance of the flora (Price Morgan et al., 2009). (f) Based on the UniFrac distance, a principal coordinate analysis (PCoA) was performed to distinguish the differences in the microbial structures of different Douchi samples.

### 2.5 Statistical analysis

In this study, R (v 4.0.0) was used to perform a Wilcox test to determine the differences in moisture content, taste index, and alpha diversity index among all groups. *p* value <.05 was considered significant. The Kruskal–Wallis test was used to calculate the difference in taste index among multiple groups. Pearson correlation analysis was used to calculate the correlation and significance between moisture content, dominant bacteria (average relative abundance is greater than 1.00%), and taste index, and the network chart was used to visualize the data. Redundancy analysis (RDA) was used to identify the different bacterial genera. Procrustes analysis (Johnson et al., 2019) was used to analyze the covariation between Douchi bacterial community and taste quality. Other drawings were done using R and Origin 2019b software.

## 3 RESULTS AND DISCUSSION

### 3.1 Taste quality analysis

Electronic tongue was used to digitally evaluate the taste indices of all Douchi samples, including the taste indices of different types and different regions, as shown in Table 1. Kruskal–Wallis test showed that there were significant differences in the taste indices among all groups (*p < *.001), indicating that there were significant differences in the taste quality of different Douchi samples (Table 1). The range of relative intensities of basic tastes such as sourness, bitterness, astringency, saltiness, and umami was greater than 1. Sourness displayed the greatest differences among the various groups, while the sourness intensity of dried Douchi from Badong County was the strongest. At the same time, we also determined the differences of each taste index between any two groups. The results showed that the samples from two regions had significant differences in other taste indexes except aftertaste‐A. In addition, there was only a significant difference in astringency between dry and wet fermented soybeans.

**TABLE 1:** Differences in taste quality of different types samples from two regions

Performance	LCD	LCW	BDD	BDW	Kruskal–Wallis
Sourness	3.08 (1.71, −1.25–11.08)^b^	0.84 (0.00, −2.02–4.53)^b^	14.34 (15.51, 6.19–21.46)^a^	12.40 (13.29, 2.38–22.07)^a^	3.90E‐05
Bitterness	−0.57 (−0.57, −1.33–0.63)^bc^	−0.88 (−1.00, −2.217–0.00)^c^	1.52 (1.38, −1.78–4.78)^a^	0.23 (−0.10, −1.24–2.88)^b^	0.00076
Astringency	0.42 (0.32, −0.35–1.58)^b^	−1.24 (−1.05, −2.86–0.00)^c^	3.08 (2.87, −2.12–9.05)^a^	0.47 (0.66, −1.95–4.48)^b^	0.00048
Aftertaste‐B	−0.39 (−0.223, −1.487–0.51)^a^	−1.48 (−1.68, −2.93–0.00)^b^	−4.14 (−4.05, −8.10–0.64)^c^	−1.36 (−1.83, −2.81–1.20)^ab^	1.50E‐05
Aftertaste‐A	−0.45 (−0.10, −2.51–1.03)^a^	−1.66 (−1.29, −3.06–0.00)^b^	−4.17 (−3.74, −8.83–1.35)^c^	−0.32 (−1.08, −1.91–2.64)^ab^	3.20E‐06
Umami	−1.80 (−1.25, −3.90 to −0.39)^a^	−0.51 (−0.76, −2.08–1.39)^a^	−5.66 (−6.35, −6.90 to −3.41)^b^	−5.62 (−6.07, −7.49 to −2.98)^b^	5.30E‐03
Richness	0.68 (0.84, −1.56–2.07)^a^	0.25 (0.14, −1.21–3.39)^a^	−2.03 (−1.88, −3.08‐−1.22)^b^	−1.73 (−1.70, −2.15 to −0.84)^b^	4.70E‐03
Saltiness	−0.30 (−1.11, −1.61–2.56)^a^	0.21 (0.00, −1.12–3.19)^a^	−6.57 (−8.45, −10.07 to −1.33)^b^	−6.77 (−8.59, −8.84 to −0.50)^b^	3.70E‐06

Note

The data presented are means (median, minimum ‐ maximum). Letters followed by different lowercase letters in the same row are significantly different to each other (*p < *.05).

To further verify our results, we analyzed the differences in taste indices according to region or type (Figure S1). The results showed that, except for aftertaste‐A, significant differences in all taste indices between the two regions, but there were no significant differences in any taste indices except astringency between the two types of Douchi. In this study, the correlation between moisture content and taste quality was further analyzed, and it was found that there was a significant correlation between moisture content and each taste index (Figure S2). This also proved that the differences in taste quality among samples were related to the region of origin, but were unrelated to the type of Douchi.

### 3.2 Composition and diversity of fungi in Douchi samples

In addition to using the electronic tongue to digitally evaluate the taste quality of various Douchi samples, MiSeq high‐throughput sequencing technology was used to analyze their fungal diversity. A total of 2,013,615 high‐quality 18S rRNA sequences were produced by high‐throughput sequencing, with an average of 50,340 sequences per sample. After dividing the sequences according to 97% similarity and removing the chimera sequences, a total of 27,721 OTUs were obtained, with an average of 711 OTUs per sample. The α‐diversity curve was used to evaluate the sequencing quality of each sample to determine whether it met the requirements of subsequent bioinformatics analysis (Figure S3). We found that the sparse curves of almost all samples did not reach the plateau stage (Figure S3a), but that the Shannon curve reached a saturation state (Figure S3b). This showed that by increasing the amount of sequencing, a small number of new species could be found, but that the microbial diversity in the samples had not noticeably changed, indicating that the current amount of sequencing is sufficient to meet the requirements of subsequent bioinformatics analysis (Wang et al., 2020). A Venn diagram was drawn based on OTU levels (Figure S3c). A total of 2,576 fungal core OTUs were found in all Douchi samples, accounting for only 3.16% of the total OTUs, but containing 1,803,640 sequences, accounting for 89.57% of all effective sequences. In addition, 201,75 OTUs appeared only once, containing 42,603 sequences and accounting for 2.12% of the total number of sequences. Although different groups of samples contain some unique OTUs, their relative content was low, indicating that there was a large number of core fungal flora in these Douchi samples.

PCoA based on unweighted and weighted UniFrac distance showed that Douchi samples from different groups showed an obvious overlap in the spatial arrangement (Figure S4a,b). At the same time, we found that there was no significant difference in the α diversity index (Chao1, Observed species, Shannon, and Simpson) of community structure in Douchi samples from different types and different regions (Figure S4c–f). This showed that there appeared to be no significant difference in the overall structure of fungi in Douchi samples among different groups.

**FIGURE 1:**
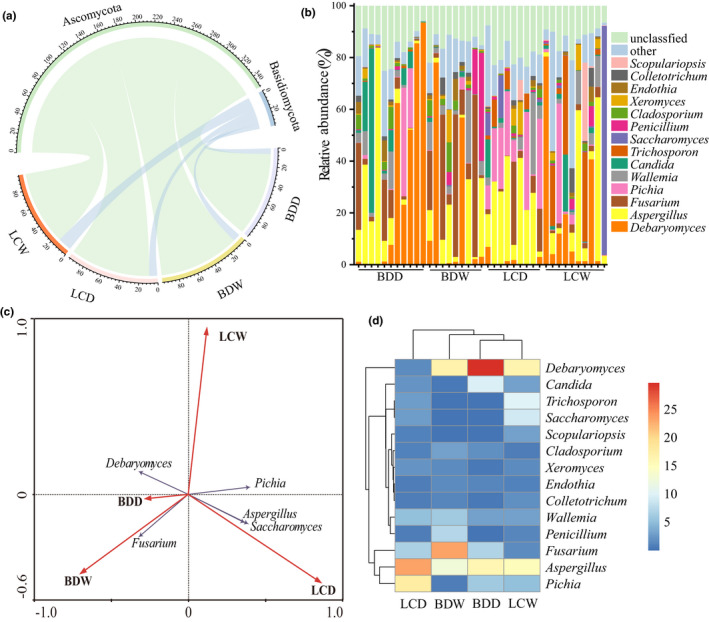
Analysis of the composition of fungal microorganisms in different Douchi samples. (a) Horizontal bar chart indicating the relative abundance of fungi at the phylum level; (b) horizontal bar chart indicating the relative abundance of fungi at the genus level; (c) bi‐ordered graph showing redundancy analysis (RDA); (d) heat maps of dominant fungi

The 18S rRNA sequencing results showed that the microbial communities of all samples covered 8 phyla, 18 classes, 37 orders, 50 families, and 72 genera. At the phylum level (Figure 1a), Ascomycota was the absolutely dominant phylum, with an average relative abundance greater than 50% and appearing in all Douchi samples, followed by Basidiomycota. It is worth noting that the average relative abundance of Ascomycota was highest in the BDD (92.57%) group and lowest in the LCW group (82.03%). In contrast, the average relative abundance of Basidiomycota in these two groups was 6.17% and 15.20%, respectively. At the genera level (Figure 1b), there were 14 fungal genera with an average relative content of more than 1.00% in Douchi, of which the average relative content of *Debaryomyces* was the highest (average: 16.87%, range: 0.0095%–93.55%). The other genera with high abundance included *Aspergillus* (16.80%, 0.019%–83.56%), *Fusarium* (9.42%, 0%–62.67%), *Pichia* (7.35%, 0.0037%–46.29%), *Wallemia* (4.34%, 0%–31.44%), *Candida* (4.13%, 0.0057%–63.41%), *Trichosporon* (3.30%, 0%–38.11%), *Saccharomyces* (3.06%, 0%–88.44%), *Penicillium* (2.28%, 0%–41.77%), *Cladosporium* (1.69%, 0.0035%–11.52%), *Xeromyces* (1.52%, 0%–12.50%), *Endothia* (1.18%, 0%–8.29%), *Colletotrichum* (1.14%, 0%–9.44%), and *Scopulariopsis* (1.02%, 0%–30.22%). Thus, there were not only some differences of the relative abundance of dominant fungi in Douchi but also important differences among different Douchi samples from the same group.

Different strains in different groups were identified by RDA (Figure 1c). The results showed that among all dominant fungal genera, *Debaryomyces*, *Fusarium*, *Pichia*, *Aspergillus*, and *Saccharomyces* were well correlated with the sample assignment on the constraint axis of the RDA sequencing diagram, suggesting that it was these five dominant fungal genera that led to important differences in the overall fungal structure of samples from different groups. In the RDA sequencing chart, *Debaryomyces* was closer to the BDD group, *Fusarium* was closer to the BDW group, and *Pichia, Aspergillus*, and *Saccharomyces* were close to the LCD group, which indicated that the *Debaryomyces* content was higher in Douchi samples of the BDD group than in samples from the other groups. On the other hand, the relative abundance of *Fusarium* in Douchi samples from the BDW group was higher than in those for other groups, while *Pichia, Aspergillus*, and *Saccharomyces* levels in samples from the LCD group are higher than in those from the other three groups. At the same time, we also counted the average relative abundance of dominant fungi in each group (Figure 1d) and the results were consistent with the results of RDA. This further proves that differences in dominant genera the main factors leading to the differences in the overall flora structure of different groups of Douchi samples.

### 3.3 Analysis of correlation between microflora and taste quality in Douchi

**FIGURE 2:**
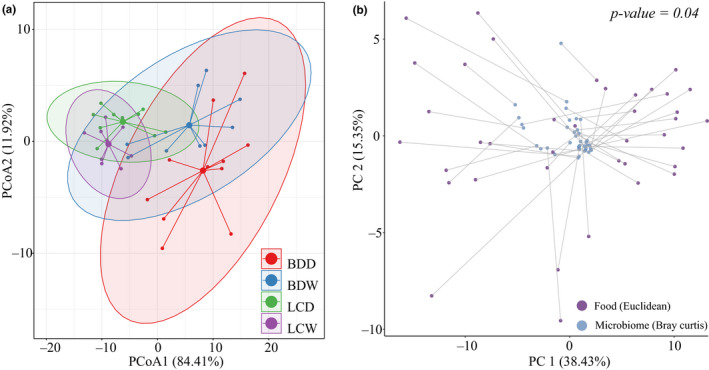
Cochange analysis. (a) Principal component analysis based on taste index; (b) Procrustes analysis

To explore the relationship between microflora and taste quality, the Procrustes method was used to analyze the cochange trend between microflora and taste quality in Douchi. First, the taste index of samples was analyzed by principal component analysis (Figure 2a). The Douchi samples of different groups showed an obvious classification trend in space, and the Douchi samples from Lichuan County gathered more closely. This showed that there were great differences in the taste quality of Douchi samples from different regions, and further proves the correctness of the conclusion in Section 3.1. Based on the principal component analysis of the floral structure and taste quality, it was found that the same Douchi sample showed obvious similarity in spatial arrangement (Figure 2b), and the correlation was very strong by Procrustes transformation and significance test (*p =* .04). This shows that the community has a significant effect on the taste quality of Douchi samples.

**FIGURE 3:**
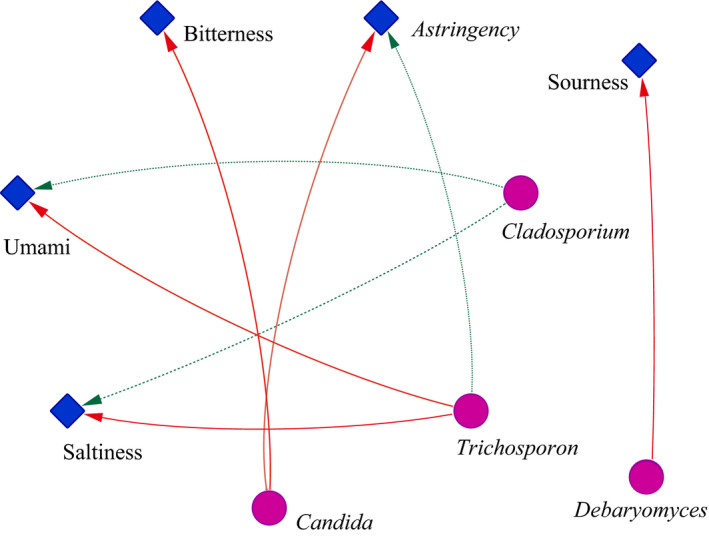
Network diagram of the correlation between dominant microorganisms and taste indexes. The width of each line represents the strength of the correlation. The wider the connection, the stronger the correlation; red indicates positive correlation, and green indicates negative correlation. The stronger the chroma, the higher the correlation

Furthermore, how the dominant fungal genus affected the taste quality of Douchi was explored (Figure 3). Results found that among the 14 dominant fungal genera, 4 fungal genera (*Cladosporium*, *Debaryomyces*, *Trichosporon*, and *Candida*) had significant effects on 5 basic flavors (sourness, bitterness, astringency, saltiness, and umami). *Cladosporium* was significantly and negatively correlated with saltiness and umami; *Trichosporon* was significantly and positively correlated with saltiness and umami, but negatively correlated with astringency; *Candida* was significantly and positively correlated with astringency and bitterness; and *Debaryomyces* was significantly and positively correlated with sourness. It has been reported that sourness and umami can be used as indicators of excellent characteristics in fermented foods, while astringency and bitterness can be used as indicators of defects in fermented foods. Furthermore, *Debaryomyces* and *Trichosporon* were conducive to the taste quality of Douchi, while *Cladosporium* and *Candida* had a negative impact on shaping the taste quality of Douchi.

## 4 DISCUSSION

In this study, we found, using Illumina MiSeq high‐throughput sequencing technology, that Ascomycota and Basidiomycota were the dominant phyla in Douchi, of which Ascomycota was the absolute dominant phylum, which was consistent with previous research reports (Lu et al., 2018). In addition, our study also demonstrated that *Debaryomyces*, *Aspergillus*, *Fusarium*, *Pichia*, and others were the dominant genera in Douchi. The average relative abundance of the dominant genera was as high as 74.10%, and related reports confirmed that these dominant genera play an important role in decomposing proteins in soybeans to produce amino acids (Wang et al., 2018). Thus, there were a large number of molds and yeasts in traditional Douchi, which can ferment proteins, starch, and sugars to produce a large number of amino acids, alcohol, carbon dioxide, and other metabolites. There were some differences between our study and previous reports on the dominant microbial species in fermented soybean products from different regions. Zhang (2018) found that two dominant fungal genera (*Pichia* and *Candida*) came from two samples, while the *Rhodosporidium* and *Yarrowia* genera appeared in a sample from Longnan. The results showed that both geography and species diversity affected the fungal species richness and diversity; Chen et al. (2015) identified fungi, yeasts, *Bacillus,* and lactic acid bacteria as the key players in Douchi fermentation, and the participation of the identified probiotic microorganisms in fermentation led to a higher quality of Douchi products. Molecular identification with the internal microbial community revealed that the microbial biomass in surface Douchi was different from that in undersurface Douchi even sampled from the same fermentation tanks, and a 10‐ to 100‐fold reduction of microbial cell counts in undersurface had been observed (Chen, 2014). The main reason for the difference in the above research results may be that the microbial community in traditional fermented Douchi is affected by many factors such as the production process, climate, sanitary environment, and temperature of the production site (He et al., 2019; Chen et al., 2017).

According to the evaluation by the electronic tongue system, there were obvious differences in the taste quality of Douchi from different regions. The sour intensity of Douchi from Badong County was significantly higher than that from Lichuan County. It is worth noting that the saltiness intensity of Douchi from Lichuan County was significantly higher than Badong County. By comparing the production process used by farmers from different areas, we found that the farmers from Lichuan County used a greater amount of salt when making Douchi, which may be the main reason for the difference in salty taste between Douchi samples from these two places. As one of the most basic seasoning, salt plays an important role in enhancing the flavor quality of food. Related reports show that salt can regulate osmotic pressure, sterilize food products, and increase appetite (Zhang et al., 2016). Jansen et al. (2003) found that *Saccharomyces rouxii* plays an important role in the fermentation of soybean products. It has the characteristics of high osmotic pressure and salt tolerance and can ferment glucose and maltose to produce ethanol, succinic acid, and other trace components, thus giving Douchi a special flavor. Osburn et al. (2018) reported that *Debaryomyces* can produce a large number of sour substances by using carbohydrates in the fermentation matrix, while Kasankala et al. (2011) has also shown that *Aspergillus* can decompose proteins to produce a variety of amino acids, thus enhancing the freshness of food and appetite. Our study also found that with the increase of salt content in Douchi, the salty taste increased, but the sour taste and fresh taste decreased significantly. It also shows that increasing the content of salt in fermented products can not only directly adjust the salty taste of food, but also further affect the sour and fresh taste of food by adjusting the structure of microflora in the fermented food.

Although there were great differences in microbial community structure in different Douchi samples, the functional expression and fermentation characteristics of the same microorganism in Douchi samples are basically the same (Wang et al., 2012). In this study, Procrustes analysis was used to find that the taste quality and the flora in Douchi samples had the same trend (Luo et al., 2020). At the same time, the results of correlation analysis showed that *Debaryomyces* and *Trichosporon* were positively correlated with the intensity of the excellent taste index of Douchi samples, while *Cladosporium* and *Candida* were positively correlated with the intensity of the defective taste index. Thus, *Debaryomyces* and *Trichosporon* play a positive role in the formation and improvement of taste quality.

This study suggests that there may be some unique fungi and even yeast groups in Douchi in different regions. On the basis of collecting, identifying, and preserving the yeast resources in Douchi in Enshi area, the whole‐genome sequencing technique can be used to analyze the fermentation and functional characteristics of yeast strains with excellent fermentation characteristics (Mixão et al., 2019), whether it is the theoretical study of Douchi microflora and genetic diversity. It is also of great significance to improve the technology and quality of Douchi. Therefore, in the follow‐up study, more different kinds of Douchi samples were collected from Gansu Province, Sichuan Province, and northern China, and the high‐throughput sequencing technique was used to realize the parallel and comprehensive analysis of fungal groups in samples from multiple provinces, in order to identify the core fungal groups in the samples from the main Douchi producing areas in China. It is also of great significance to verify the conjecture that there are unique fungi and even yeasts in Douchi in different regions. It is worth noting that due to the characteristics of MiSeq sequencing, it cannot be used at the "species‐level" in the analysis of microbial community structure, which is a limitation of our study of fungi in Douchi. Therefore, in follow‐up research, it will be extremely critical to further analyze the microbial community in Douchi by using the technology of metagenome sequencing or the third generation sequencing in a higher taxonomic position (Heil et al., 2018; Tedersoo and Anslan, 2019).

## 5 CONCLUSIONS

Electronic tongue technology was used to digitally evaluate the taste quality of Douchi samples, combined with high‐throughput sequencing technology to identify the fungal community structure in Douchi samples, to analyze the effect of fungal flora on taste quality. We found that there were great differences in the taste quality of samples from different regions, while different types of Douchi from the same regions essentially tasted the same. Sourness, umami, richness, and saltiness were the main reasons for the differences between the two regions, while the high‐throughput sequencing results showed that there were important differences in dominant fungi among different types of Douchi from different regions, and *Debaryomyces, Fusarium, Pichia, Aspergillus*, and *Saccharomyces* were the main dominant fungal genera that caused these differences. Meanwhile, the results of correlation analysis showed that *Debaryomyces* and *Trichosporon* are conducive to the formation of the taste quality of Douchi, while *Cladosporium* and *Candida* negatively impact the taste quality of Douchi.

## CONFLICTS OF INTEREST

The authors declare that they have no conflicts of interest.

## Supporting information

Fig S1

Fig S2

Fig S3

Fig S4

## Data Availability

All sequence data from this study have been submitted to the MG‐RAST database with the entry number mgp95948 (https://www.mg‐rast.org/mgmain.html?mgpage=metazen2&project=mgp95948).
